# How Does Topography Affect the Value of Ecosystem Services? An Empirical Study from the Qihe Watershed

**DOI:** 10.3390/ijerph191911958

**Published:** 2022-09-22

**Authors:** Li Li, Yonghui Li, Lan Yang, Ying Liang, Wenliang Zhao, Guanyu Chen

**Affiliations:** 1 School of Government, Beijing Normal University, Beijing 100875, China; 2 Henan Provincial General Institute of Urban and Rural Planning and Design, Zhengzhou 450044, China; 3 School of Surveying and Planning, Shangqiu Normal University, Shangqiu 476000, China

**Keywords:** ecosystem service value, land-use/cover change, topographic position index, sensitivity index, Qihe watershed

## Abstract

Topographic position indices (TPIs) measure essential impacts on ecosystem service supply capacity. The identification of changes in ecosystem services and value metrics under varying TPIs has become a topical subject of global change research. Multidimensional changes in spatiotemporal and geographical aspects of ecosystem service values (ESVs) are assessed in this article using land cover/use data from 2000–2015. Effects of land-use/cover changes and topographic indices on ESVs are explored using the Chinese terrestrial unit area ecosystem service value equivalence table combined with topographic factors. A sensitivity index is introduced to quantify the robustness of total ESV to land-use/cover and topographic indices. The results show that: (1) The total ESV in the Qihe watershed declined with a change in land-use/cover during the period 2000–2015. The maximum ESV was CNY 1.984 billion in 2005 and the minimum was CNY 1.940 billion in 2010; (2) The response of ESV to land/use cover varied greatly across TPIs, with the most significant change in ESV occurring in the 0.6–0.8 TPI range and the greatest change in a single ecosystem service occurred in water areas; (3) The sensitivity indices of ESVs are all less than 1. The sensitivity indices of unused land and water tended to zero. Woodland sensitivity indices were the highest at 0.53, followed by those of arable land and grassland, owing to the large proportion of arable land and grassland areas in the overall area of land-use categories.

## 1. Introduction

Complex topography typically offers a variety of ecosystem services and significant spatial heterogeneity across watersheds. These services often include biodiversity conservation, water supply, food production, and soil conservation [1,2,3,4,5]. Nonetheless, the integrated measurement of ecosystem service capacity and regional differences between watersheds has presented a research challenge for geographers, ecologists, and economists [6,7,8,9,10].

Ecosystem Service Value (ESV) research was pioneered by Constanza in 1997 [11,12,13,14]. Ouyang et al. [15,16] and Xie et al. [17,18], among other prominent Chinese scholars, quickly followed up with an assessment of the capacity and value of ecosystem services in China. Using the global ecosystem services assessment by Constanza, Xie et al. [19] established a Chinese terrestrial ecosystem services assessment system. The value of five ecosystem service functions (1. Preserving the equilibrium of O_2_ and CO_2_ in the atmosphere; 2. Aiding water conservation; 3. Conserving total organic matter; 4. Providing nutrient storage and cycling; 5. Providing a purifying effect on the environment) in China was estimated by Ouyang et al. [20] using alternative engineering, shadow pricing, and profit and loss analysis.

The Millennium Ecosystem Assessment (MEA) demonstrated that the capacity and the value of global ecosystem services are largely underestimated, and that an accurate estimate of the capacity and value of ecosystem services can improve land-use science [21]. Simultaneously, the MEA proposed that ecosystem services research should evolve from the current single static value assessment to the assessment of the ecosystem services’ impact on human well-being, including concepts such as regional variability, multi-scale ecosystem services, and the dynamic evolution of ecosystem services [22,23].

Owing to these developments, the valuation of ecosystem services has become a high priority topic in ecosystem services research, especially research focusing on the impact of changes in ecosystem services in the context of global change and including the consideration of human activities on regional sustainable development [24,25]. In this context, many ecosystem service payment projects have been implemented in watersheds around China and are providing a basis for government policies on ecological protection [26,27]. Quantitative investigations of anthropogenic influences on the ESV, focusing on land-use/cover change, are becoming popular [28,29,30,31]. Nonetheless, there remains a lack of scientific standards for applying scientific rigor to ecosystem services in regional development and ecological conservation, making it difficult to operationalize in regional development planning.

Yang et al. analyzed the trade-offs between ecological health and socioeconomic development in 2040 under different land-use scenarios, by using multi-temporal, high-resolution (0.5 m) remote sensing satellite imagery and biophysical models, setting a precedent for the practical application of ecosystem service analysis [32]. Regional variability and dynamic changes in ecosystem services are defined by human activities with land-use at their center, dramatically influencing the structure, processes, and function of the ecosystem. Understanding the multidimensional patterns of ecosystem service changes and influencing variables at the local watershed scale bear practical implications for land resource management and human well-being enhancement [33,34,35,36].

At present, most research has focused on the quantitative analysis of a single time node and a single type of service capacity in a region, whereas the trade-off synergies and geographical and temporal differences of numerous service capabilities have received insufficient attention [37]. The equivalent factor method has the advantage of visualizing changes in ecosystem services and requires fewer parameters, so it is often used to estimate the value of ecosystem services [18]. Studies have been conducted to assess the ESV at different scales such as for provincial scales [38], mountainous regions [39], and watersheds [40], and to estimate the ESV from different land-use types such as glaciers [41], forests [42], grasslands [43], and wetlands [44]. However, little research has been conducted on multidimensional variations in ESV at the small watershed scale in combination with topographic features [9,45].

Small watersheds are basic and complete natural geographical units, and their complex geomorphological types render them capable of a variety of ecosystem service functions (biodiversity conservation, water supply, production, regulation, etc.). It is vital to research the spatial–temporal variability of ESV in small watersheds for human well-being. However, there are few studies that incorporate the multidimensional analysis of spatial–temporal variability of ecosystem service in small watersheds with topographic gradient effects. Consequently, determining ways to evaluate the spatial–temporal variability of ESV in connection with topographic features has emerged as a critical issue in this study. Given that ecosystem services are characterized by regional heterogeneity and dynamic changes, especially resulting from human activities centered on land use, the structure, processes, and function of an ecosystem can change significantly. Thus, it is of practical significance to understand the multidimensional change patterns and influencing factors of ecosystem services at small watershed scales to facilitate the rational use of land resources and the improvement of human well-being.

The Qihe watershed is located in the transition area between the second and third steps in China, between the Taihang Mountains and the North China plain. This watershed serves an important water-conservation role, and the terrace transition zone is distinguished by its peculiar geographic relief. From 2000 to 2015, the ESV in this region was evaluated using land-use/cover data and a coefficient-corrected terrestrial ecosystem unit area scale was developed. Within the watershed, the total ESV, individual ESVs, and sensitivity indices were calculated. The topographic position of individual ESVs was also evaluated to further investigate the impacts of land-use/cover and identify spatial differentiation patterns on ESV in a small watershed.

The main objective of this study is to reveal how topography affects the spatial and temporal distribution of ESV in a mountain-plain transition zone. We have two specific questions: (1) What are the spatial–temporal characteristics of ecosystem service values in the Qihe watershed? (2) How do topographic features affect the ESVs? To answer these questions, we first corrected China’s terrestrial value ecosystem service equivalence table using grain prices and production in Henan Province. Secondly, we analyzed the differences in spatial–temporal ecosystem services. Finally, we used the topographic position index (TPI) and a sensitivity index to investigate the characteristics of the regional ESVs. 

## 2. Data Sources and Research Methods

### 2.1. Study Area

**Figure 1:**
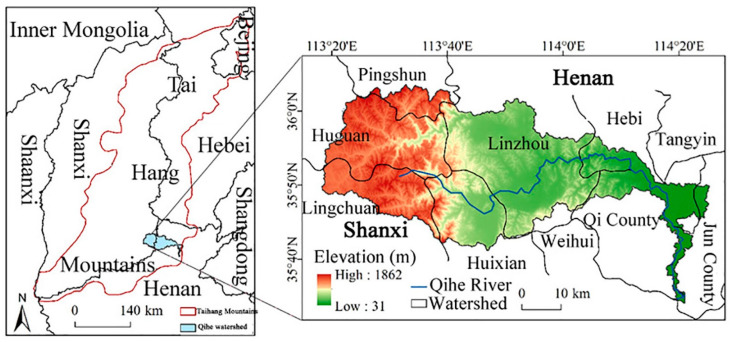
Location and elevation of the study area.

The Qihe watershed (35°32′–36°15′ N, 113°15′–114°23′ E) is located between the southwestern part of the North China Plain and the southern part of the Taihang Mountains. The Qihe River originates from the Fangnaoling mountains in Lingchuan county, Shanxi Province. It then flows through the Henan Province into the Weihe River, a tributary of the Haihe River. The watershed area is 2227 km^2^, and the elevation trend is from high in the west to low in the east (Figure 1). The main climate type is a warm temperate semi-humid continental monsoon climate, with an average annual precipitation of 574 mm and an average annual temperature of 11.9 °C. Complex topography renders the ecological environment of the Qihe watershed fragile, and diverse landform types present complex variations in ecosystem services within the basin.

### 2.2. Data Sources and Initial Data Processing

Land cover/use data from the Qihe watershed (2000–2010) were obtained from the China Earth System Data Sharing Platform-Middle and Lower Yellow River Scientific Data Center (http://www.geodata.cn/). Based on LANDSAT multi-band remote sensing images (from Geospatial Data Cloud, http://www.gscloud.cn/), 2015 land-use data were interpreted visually using human–machine interaction and surveyed in the field employing historical land-use maps of the study area, with a kappa coefficient of 86%. Digital elevation model (DEM) data were obtained from the Geospatial Data Cloud (http://www.gscloud.cn/). The land-use data were all in the form of 1:100,000 vector data and the raster data were in a uniform grid format with a spatial resolution of 30 m. The geographic coordinate system used was WGS_1984_Albers. The socio-economic data used in the study were obtained from the Henan Provincial Statistical Yearbook (2000–2015) and the China Statistical Yearbook (2000–2015). 

We referred to the research methods of Xie et al. [17,18,19] who excluded construction land in this study area from their estimation of ESV. A coefficient correction of the Chinese terrestrial ESV per unit area scale was performed using food production and arable prices in Henan province. This model was used to estimate the value and change trend of five major ecosystem services categories (arable land, woodland, grassland, water area, and unused land) from 2000 to 2015.

A TPI was used to evaluate the shift in total and individual ESV. A 5 km × 5 km grid was constructed in ArcGIS 10.3 and the different land-use types on the grid were multiplied by ESV coefficients, and then divided by the grid-cell area to obtain ESV densities. Changes in total ESV and individual ESV in relation to the TPI were calculated separately using land-use type area changes. The ESVs in relation to TPIs were calculated and spatially differentiated by utilizing a fishing-net function [46]. 

### 2.3. Methodology

**Figure 2:**
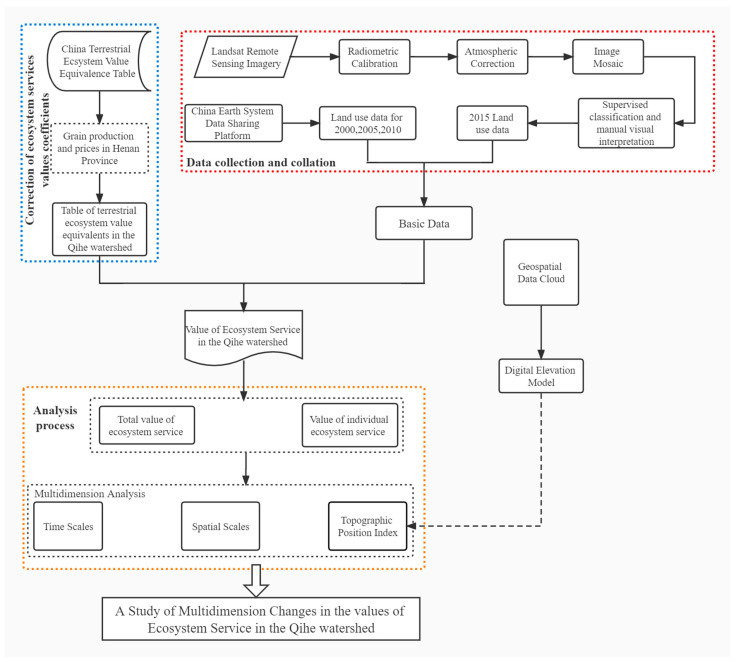
The research framework used for studying multi-dimensional (spatial, temporal, and TPI) changes in the ESV of the Qihe watershed.

The ESV of the Qihe watershed was investigated using the research framework for the study of ESV multidimensional changes (spatial, temporal and TPI) summarized in Figure 2. The framework consisted of three main components:

(1) Data preparation: In 2015, land-use data from the Qihe watershed were obtained using human–computer interactive visual interpretation and field survey of remote sensing images, based on reference to land-use maps of previous years. Additionally, land-use data for 2000, 2005, and 2010 were downloaded from the China Earth System Data Sharing Platform—Middle and Lower Yellow River Scientific Data Center. Socio-economic data and other relevant data were extracted from the China Statistical Yearbook (2000–2015) and Henan Provincial Statistical Yearbook (2000–2015). DEM data were obtained from the Geospatial Data Cloud Platform.
(2) Ecosystem service value accounting: The existing China terrestrial ecosystem services table could not be directly applied to the calculation of regional ESV. Consequently, its parameters were corrected using grain production and prices from Henan Province. Further integration of historical land-use data was then performed to estimate the value of ecosystem services in the years 2000, 2005, 2010, and 2015.
(3) Multi-dimensional change analysis of ESV: A comprehensive analysis of the changes in the total ESV and individual ESVs in three dimensions (i.e., spatial, temporal, and TPI) was performed.

#### 2.3.1. Estimating the Value of Ecosystem Services

As noted above, the value coefficients per unit area of the terrestrial ecosystem in China were modified in this study. Grassland, forest, cropland, desert, and watershed in the new system correspond to grassland, woodland, arable land, unused, and water in the original system, respectively [7,8,9]. The Qihe watershed area in Henan Province spans 1424 km^2^, accounting for 64% of the total area. The average grain yield of 5305.24 kg/hm^2^ and the grain price of 1.36 CNY/kg in Henan Province from 2000 to 2015 were used to correct the table of the terrestrial ESVs [46].

The value of food production per unit area is given by the formula:

(1) $$Va=\frac{1}{7}\sum_{m=1}^{n}\frac{a_{m}p_{m}q_{m}}{A}$$

where *m* refers to the type of crop, with *m* = (1, 2, 3,···, *n*); *Va* denotes the economic value of food production function per unit area of the arable ecosystem; *p_m_* is the average price of the *m* food crop; *q_m_* is the yield per unit area of the *m* crop; *a_m_* is the area of the *m* crop cultivation; *A* is the area of food cultivation. *V_ij_* is defined as follows:

(2) $$V_{ij}=e_{ij}v_{a}$$

where *V_ij_* is the service value of ecosystem service *i* in ecosystem *j* per unit area; *e_ij_* is the equivalent factor of the service value of ecosystem service *i* in ecosystem *j* in the study area; *i* is the ecosystem service type, with *i* = (1, 2, 3,···, *n*), and *j* is the type of ecosystem.

According to Equation (1), at constant prices in 2015, the ecosystem service value of food production per unit area of arable land was calculated as 1030.73 CNY/ha. We refer to Xie et al. [18,47] and Ouyang et al. [15,16] for other land-use types (Table 1). 

#### 2.3.2. Single Land-Use Dynamic Approach

The single land-use dynamic approach was introduced to measure the quantitative change characteristics of a land-use type over a set time horizon in the watershed [48]. The calculation formula used is as follows:

(3) $$K=\frac{U_{b}-U_{a}}{U_{a}}\times \frac{1}{F}\times 100\%$$

where *U_a_* is the area of land-use type *a* at the beginning of the period; *U_b_* is the area of the same land-use type at the end of the period; *F* denotes the study period; and *K* is the annual rate of change during the study period.

#### 2.3.3. Topographic Position Index (TPI)

The TPI was introduced to reflect the multidimensional changes in the ESV within the watershed along topographic gradients, and to characterize the spatial pattern distribution of ESV [49]. The calculation formula is as follows:

(4) $$T=\log_{10}\left(\left|\frac{E}{E_{mean}}+1\right|\times \left|\frac{D}{D_{mean}}+1\right|\right)$$

where *E* is the elevation value of the raster; *E_mean_* is the average elevation value of the raster; *D* is the slope value of the raster; *D_mean_* is the average slope value in the raster; *T* is the topographic position index. The magnitude of *T* is affected by both the elevation value and the slope of the study area. If the elevation is larger and the slope is steeper, *T* is larger, and vice versa.

#### 2.3.4. Sensitivity Analysis of Ecosystem Service Values

In this paper, a Coefficient of Sensitivity (CS) was introduced to test the effects of land-use change on ESV, and to discern the dependence of ESV on the value coefficients derived from 2000 to 2015 [9]. If CS > 1, this reveals that the change in the ESV coefficient of one land-use type has a significant impact on the total ESV. If CS < 1, this can indicate that the change in the ESV in one land-use type does not have a significant impact on the ESV in the entire study area per unit area. The CS was defined as follows:

(5) $$\mathrm{CS}=\left|\frac{\left({\mathrm{ESV}}_{j}-{\mathrm{ESV}}_{i}\right){/\mathrm{ESV}}_{i}}{\left({\mathrm{VC}}_{\mathrm{jk}}-{\mathrm{VC}}_{\mathrm{ik}}\right){/\mathrm{VC}}_{\mathrm{ik}}}\right|$$

where ESV is the total ESV of the study area (CNY); VC is the ESV coefficient of each land-use type (CNY/hm^2^); subscript k refers to the land-use type; subscripts i and j refer to before and after the adjustment of the ESV coefficient, respectively.

## 3. Results

### 3.1. Land-Use/Cover Changes in the Qihe Watershed

The land-use/cover types in the Qihe watershed are mainly grassland, arable land, and woodland, with a smaller area of watershed and unused land. Land-use/cover changed significantly during the study period (Table 2). The area of construction land, watershed, and unused land increased. Forest land area first increased and then decreased, grassland area decreased and then increased, while arable land area continued to decrease. The land-use single dynamic approach demonstrated that unused land was the highest, followed by watershed, and woodland was the smallest. 

### 3.2. Changes in the Value of Ecosystem Services in the Qihe Watershed

#### 3.2.1. Temporal Change

**Figure 3:**
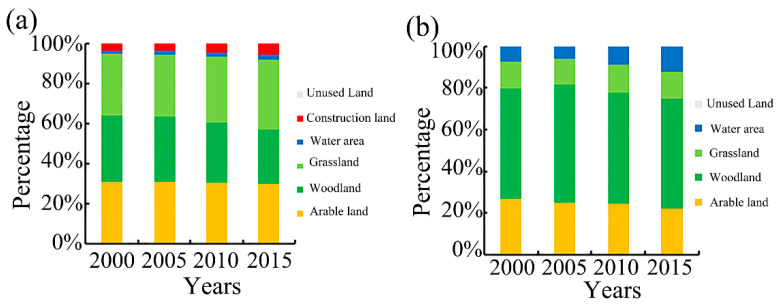
The percentage of land-use/cover by area (**a**) and by ecosystem services value (**b**) in the Qihe watershed.

Each land-use type ESV was obtained by multiplying various land-use type areas at different periods with the corresponding ESV coefficients. The highest share of woodland ESV in total (53%) from 2000–2015 is shown in Figure 3. It is clear that the grassland areas account for 36% of the total (Figure 3a), but the ESV percentage is less than 14% (Figure 3b). The reason for this is that the ESV coefficients of both water and woodland land-use are greater than the ESV coefficient of other land-use types [18].

The total ESV in 2000 was CNY 1.954 billion (Table 3), and mainly composed of arable land, woodland, and grassland ESVs. The total ESV of the study area increased by CNY 1.981 billion in 2005. The CNY 0.66 million increase in woodland ESV accounted for the major part of the gain and compensated for the CNY 0.36 million decrease in arable land ESV.

Between 2000 and 2005, the land-use type with the most significant decrease in ESV was arable land (5110.72 ha), with the largest increase in woodland (4370.37 ha). The total ESV decreased by CNY 25 million between 2000 and 2010, because woodland and arable land were converted to other land-use types. The increase in water and grassland areas compensated for the decrease in total value. The largest increase in land-use type was in water area. Compared to the period 2000–2005, the total ESV of the water land-use type decreased at a high rate of change (1.9%), and the ESV showed an increasing and then decreasing trend.

During the 2000–2015 period, the total ESV decreased to CNY 1.942 billion, with the decrease in area of arable land and woodland being the main reason for the decrease in the total ESV. The total ESV underwent an increase of CNY 27 million from 2000 to 2005, a decrease of CNY 52 million yuan from 2005 to 2010, and an increase of CNY 11 million from 2010 to 2015. The decrease in the Qihe watershed total ESV is mainly attributed to the decrease in areas of arable land and woodland, and increase in the area of construction land-use.

Each ESV and its contribution rate from 2000 to 2015 were summarized using the secondary type value coefficients multiplied by the corresponding land-use type for each calendar year (Table 4). The different ecosystem function ESVs exhibited small variations, with the highest contribution of 18.83% from WD and the smallest contribution from EC (3.99%). The ranking of the individual ESVs is as follows: WD > SFC > WC > BC > CR > GR > FP > RM > EC.

#### 3.2.2. Spatial Variation

Based on land-use/cover data from 2000, 2005, 2010, and 2015, different ESV land-use types in the four years were calculated for each grid cell, as well as the region’s total ESV [37]. The value density was classified into five classes (0–1000 CNY/km^2^, 1000–2000 CNY/km^2^, 2000–4000 CNY/km^2^, 4000–7000 CNY/km^2^ and >7000 CNY/km^2^) by referring to the study of Xu et al. [48]. The amount of ESV density change was divided into six categories (<−4000 CNY/km^2^, −4000 to −1000 CNY/km^2^, −1000–0 CNY/km^2^, 0–2000 CNY/km^2^), displaying a clear reflection of the difference in spatial distribution and ESV change trend [50]. 

**Figure 4:**
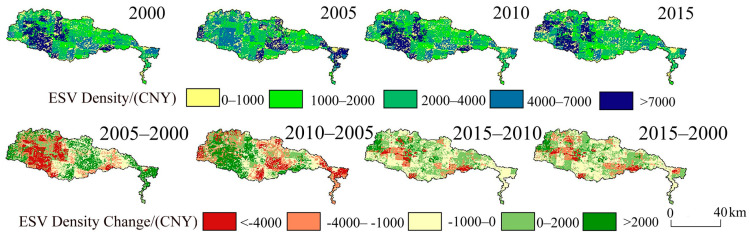
Spatial distribution and change in ecosystem service value density in the Qihe watershed from 2000 to 2015.

The overall ESV in the Qihe watershed was high in the southwest and low in the northeast (Figure 4). ESV densities > 7000 CNY/km^2^ were mainly distributed in areas covered by woodlands and grasslands in the upper reaches of the watershed. Densities of 4000–7000 CNY/km^2^ were mainly distributed in the central part of the watershed covered by cropland and grassland. The regional ESV density of grassland cover was between 2000–4000 CNY/km^2^ and was the most widely distributed, while the ESV density in the middle and lower reaches of the watershed was <2000 CNY/km^2^, displaying a fragmented distribution. During the period spanning 2000–2005, the spatial ESV distribution density was diminished in the upper reaches of the watershed and increased in the middle and lower reaches. The most significant decrease in ESV in the upper reaches was caused by the rapid expansion of woodland reclamation into arable land and construction land. At the same time, the expansion of the water area caused an increase in ESV density in the middle and lower reaches, leading to a gradual improvement in habitat quality in the middle and lower reaches of the basin [51]. The most obvious change in ESV density between 2005 and 2010 was in the lower reaches due to the growth in construction land area and reduction in grassland and arable land area. As different land-use types correspond to various ESV coefficients, a land-use type shift in the watershed will cause a corresponding change in its ESV. It is clear that during the study period, the ESV in the Qihe watershed was in a dynamic process of change. The decrease in woodland and grassland areas, and the rapid expansion of construction land explained the most obvious changes. Overall, the total ESV showed a decreasing trend.

### 3.3. Analysis of the TPI of ESVs

**Figure 5:**
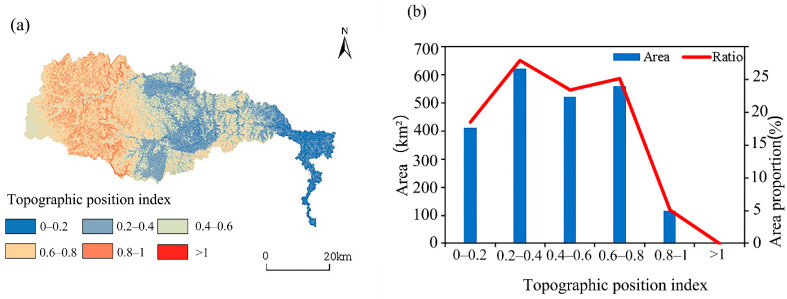
Topographic position index distribution map (**a**) and land-use type area by different topographic position index (**b**).

Referring to research by Chen et al. [44], the TPI was classified into six levels (0–0.2, 0.2–0.4, 0.4–0.6, 0.6–0.8, 0.8–0.1 and >1). The TPI is high in the east and low in the west (Figure 5a). TPI values from 0–0.2 are mainly distributed in the lower reaches of the watershed; 0.4–0.6 TPI are distributed in the middle reaches, and 0.8–1 TPI are found in the upper reaches of the basin. Figure 5b demonstrates that land-use area is mainly distributed on 0.2–0.4 and 0.6–0.8 TPI, which account for 27% and 25%, respectively. Values of TPI > 1 have the least distributed area (0.22 km^2^) and the smallest ratio (0.01%). Overall, the TPI < 1 is distributed most widely in the Qihe watershed, accounting for 99.9% of the area.

#### 3.3.1. Topographic Factor Analysis of ESV Change

**Figure 6:**
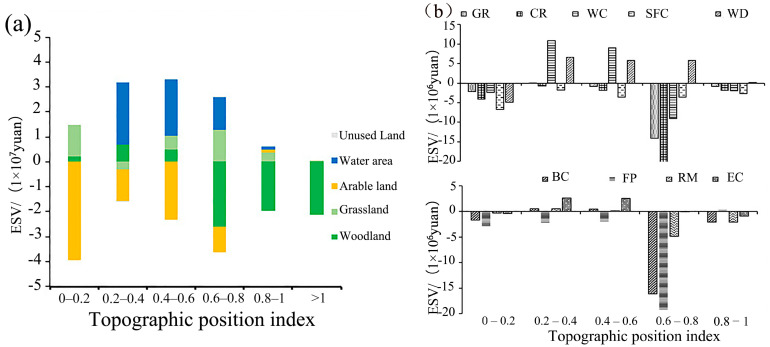
Changes in total ESV (**a**) and individual ESVs (**b**) in the Qihe watershed by topographic position index from 2000 to 2015.

The land-use/cover in 2000 and 2015 were used to analyze the change in ESV and individual ESVs in relation to TPI (Figure 6a). The most significant decrease in arable land ESV (CNY 39.52 million) occurred within the 0–0.2 TPI range, while grassland and woodland ESV increased by CNY 12.57 million and CNY 2.34 million, respectively, mainly caused by low-value TPI areas being highly influenced by human activities [29].

A significant increase in watershed ESV (CNY 25.22 million) and an increase in woodland ESV of CNY 6.85 million occurred within TPIs of 0.2–0.4. The high ESV coefficient of water areas was the major factor behind the significant increase in water ESV, while arable land and grassland ESV decreased by CNY 12.5 million CNY and CNY 3.2 million CNY. Small overall changes in the ESV of areas with TPI from 0.4 to 0.6 were due to increases in water, grassland, and woodland ESVs and decreases in arable land ESV. Woodland ESV decreased in areas with high TPI (i.e., TPI > 0.6), with the largest reduction being within the 0.6–0.8 TPI range (CNY 25.89 million). 

As shown in Figure 6b, the individual ESVs vary across TPI values. For example, the CR ESV decreased by CNY 21.14 million, FP ESV decreased by CNY 19.25 million, BC ESV decreased by CNY 16.14 million, GS ESV decreased by CNY 14.13 million, and WC decreased by CNY 9.01 million, which were mainly due to the largest reduction in a woodland area during this interval. The WC ESV increased by CNY 10.98 million and CNY 9.07 million within the 0.2–0.4 TPI and 0.4–0.6 TPI intervals, respectively. Table 1 and Table 2 indicate that the large water area and high WC ESV coefficient are the main reasons for the watershed ESV increase.

#### 3.3.2. Spatial Characteristics of TPI of Ecosystem Service Value Change

**Figure 7:**
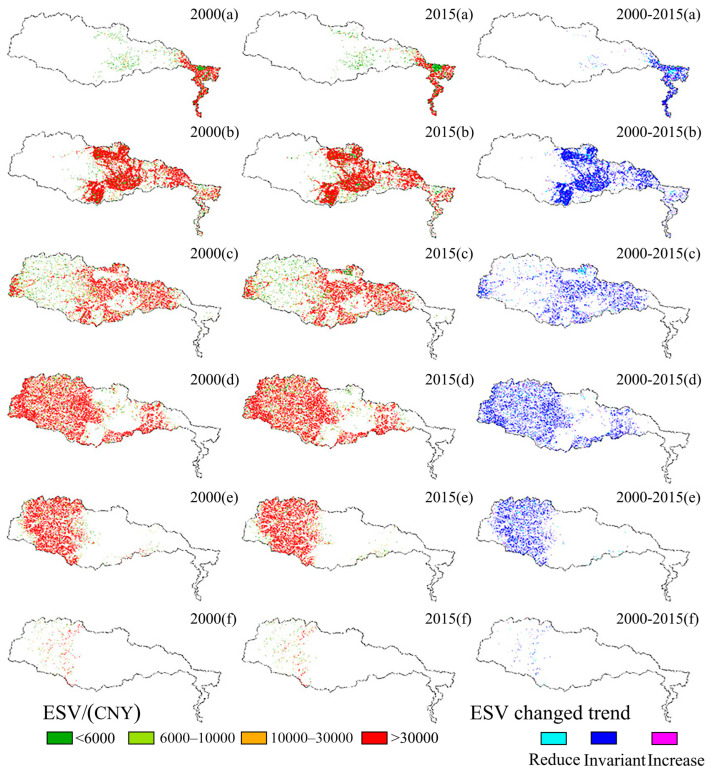
Characteristics of change in ecosystem service values of different topographic position indices (Note: a, b, c, d, e and f indicate 0–0.2, 0.2–0.4, 0.4–0.6, 0.6–0.8, 0.8–1, TPI > 1) in the Qihe watershed from 2000–2015.

Here, we use the land-use/cover data from the two years 2000 and 2015 and combine them with the TPI analysis to investigate the dynamic change process of ESV. By referring to the work of Li et al. [22], the ESV was divided into <6000 CNY, 6000–10,000 CNY, 10,000–30,000 CNY, and >30,000 CNY in total, with topographic position indices of 0–0.2, 0.2–0.4, 0.4–0.6, 0.4–0.8, 0.8–1 and >1 (Figure 7). The results suggest that ESV greater than CNY 30,000 in 2000–2015 was distributed over a large area and concentrated within TPIs of 0.2–0.4, 0.6–0.8, and 0.8–1. ESV < 6000 CNY was mainly distributed within 0–0.2 and 0.4–0.6 TPI grading, suggesting that ESV is higher and widely distributed in 0.2–0.4, 0.6–8, and 0.8–1 TPI, primarily due to the wide distribution area of grassland and woodland and the higher ESV coefficients of these two types. The TPI > 1 accounts for a small proportion of the area, and the distribution of grassland and woodland in this zone was small, thus the ESV distribution is not significant and located within two intervals of CNY 6000–10,000 and CNY 10,000–30,000. There was a small change in land-use types resulting in the change in ESV from 2000 to 2015. The construction land area increase had no direct effect on the total ESV, while the increase in water area and grassland by 1998.83 ha and 8854.38 ha, respectively, compensated to some extent for the total ESV loss caused by the arable land area decrease. 

### 3.4. Sensitivity Analysis of the ESVs in the Qihe Watershed

The modified ESV coefficients of the Qihe watershed were adjusted up and down by 50%, respectively, to calculate the total ESV for all years, and to estimate the sensitivity of the results to this value (Table 5) [9]. The calculated results of the adjusted ESV coefficients for each land-use type indicate a sensitivity index of less than 1. The CS of unused land and water tends to zero, reflecting the inelasticity of the total ESV concerning the service value coefficient, demonstrating the reliability of the results in this paper. The large ESV coefficient of woodland land led to the highest sensitivity index (about 0.53), followed by arable land and grassland, owing chiefly to the large proportion of arable land and grassland area in the total. Both the small area of unused land and low ESV coefficient resulted in the lowest sensitivity index for unused land (0.0005). During the period 2000–2015, the CS of grassland, water, and unused land showed a stable and then increasing trend. The CS of arable land displayed a gradual decrease, the woodland CS displayed an increase and then a decrease, and their CS changes were consistent with the changes in their respective adjusted areas. Overall, the ESV sensitivity index indicates that the ESV coefficients of various land-use types still bear many uncertainties, but the total ESV in the Qihe watershed remains in a stable state.

## 4. Discussion

The equivalent factor method used here to estimate the ESV in the Qihe watershed can visually reflect the change in ESV. The advantage of the equivalent factor method is its lower data demand compared with the price per unit area of the service function method, which is suitable for the study of ESV at regional and global scales [20]. The changes in land-use during the research period had a profound impact on the ESV, with the changes acting as a guide for adjusting the land-use structure and optimizing the land-use pattern. Topographic elements have a significant impact on regional land-use patterns and spatial structure. Therefore, investigating the dual response of ESV to land-use/cover and topographic factors can be a useful method for assessing the quality of the ecological environment in a watershed. Analyzing the interaction between individual ESV and topographic factors plays an important role in enhancing human well-being and building harmonious habitat relationships. The purpose of this paper is to provide a reference for small watershed-scale ecosystem service research and ecological environment construction.

## 5. Conclusions

The study conclusions are as follows:

(1) The land-use types in the Qihe watershed from 2000 to 2015 were mainly arable land, forest land, and grassland, the sum of which accounts for more than 90% of the total area. The land-use/cover changes were obvious as the areas of cultivated land and forest land decreased by 12,971.61 ha and 2104.05 ha, respectively, and the areas of grassland and water increased by 8854.38 ha and 43,234.8 ha, respectively.
(2) The ESV in the Qihe watershed decreased by CNY 0.14 billion from 2000 to 2015. During the study period, the total ESV increased, then decreased, and then increased again. The highest ESV occurred in 2015, with a value of CNY 1.981 billion. The contribution level of each individual ESV remained stable, with waste treatment exhibiting the highest contribution level of 18.84%, followed by soil formation and protection.
(3) There was a significant influence of topography on the ESV. The largest decrease of CNY 39.52 million in cropland ESV and the largest increase of CNY 12.56 million in grassland ESV occurred within the 0–0.2 TPI range. The largest increase in the 0.2–0.4 TPI range was that of water ESV (CNY 25.19 million) and the largest decrease in the 0.6–0.8 TPI range was that of grass ESV (CNY 25.89 million). The largest reductions in individual ESVs were observed in the 0.6–0.8 TPI range. The ESV of water supply increased by CNY 10.98 million and 9.07 million within the areas of TPI in the 0.2–0.4 and 0.4–0.6 intervals, respectively.
(4) The sensitivity index of the ESV in the Qihe watershed is less than 1. This implies a certain lack of elasticity for the value coefficient and characterizes the robustness of the research results in this paper.

The ESV of the Qihe watershed was estimated using the Chinese terrestrial unit area ecosystem service value scale. Additionally, the spatial and temporal evolution characteristics from the period 2000–2015 were analyzed. Quantitative studies of the ESV in this watershed bear insufficient explanatory power for the trade-offs and synergistic relationships between ecosystem service functions. In future research, the trade-offs and synergistic relationships of ecosystem service functions within the Qihe watershed will be refined based on this paper.

## Figures and Tables

**Table 1:** Table of ecological service value equivalents per unit area of terrestrial ecosystems in the Qihe watershed (CNY/ha) as used in this study area.

Type I	Type II	Arable Land	Woodland	Grassland	Water Area	Unused Land
Adjustment Services	Gas Regulation (GR)	515.37	2196.22	88.60	0.00	0.00
	Climate Regulation (CR)	917.35	1770.13	219.42	407.00	0.00
	Water Conservation (WC)	618.44	2078.02	220.25	18,033.2	26.50
Support Services	Soil formation and conservation (SFC)	1504.87	2575.16	353.56	8.80	17.70
	Waste Disposal (WD)	1690.40	1419.60	1227.92	16,086.60	8.80
	Biodiversity Conservation (BC)	731.82	2195.01	580.43	2203.30	300.80
Supply Services	Food Production (FP)	1030.73	462.67	802.33	88.50	8.80
	Raw Materials (RM)	103.07	1601.40	4.97	8.50	0.00
Cultural Services	Entertainment Culture (EC)	10.31	833.94	93.56	3840.20	8.80
	Total	7122.36	15,132.15	3591.04	40,676.10	371.40

**Table 2:** Land-use/cover change (ha) and percentage (%) in Qihe watershed from 2000 to 2005.

Time	Category	Arable Land	Woodland	Grassland	Waters Area	Construction Land	Unused Land
2000	Area (ha)	73,417	68,287.3	69,438.7	3596.49	7893.35	10.9705
2005		68,306.28	72,657.67	69,415.12	3603.19	8737.88	10.971
2010		66,183.28	67,930.32	73,540.46	4063.92	10,932.70	79.67
2015		60,445.36	66,183.25	78,293.08	5595.32	12,128.45	80.32
2000–2005	Area change (ha)	−5110.72	4370.37	−23.58	6.70	844.53	0.00
2005–2010		−2123	−4727.35	4125.34	460.73	2194.82	67.61
2010–2015		−5737.92	−1747.06	4752.61	1531.4	1195.75	0.69
2000	Percentage (%)	32.97	30.67	31.21	1.62	3.52	0.01
2005		32.62	30.67	31.16	1.62	3.92	0.01
2010		29.71	30.5	33.02	1.82	4.91	0.04
2015		27.14	29.72	35.15	2.51	5.44	0.04
2000–2005	Single-motion Attitude (%)	−1.39	1.28	−0.01	0.01	2.14	0.01
2005–2010		−0.62	−1.30	1.19	2.56	5.02	123.2
2010–2015		−1.73	−0.51	1.29	7.54	2.19	0.17

**Table 3:** Change amount (CNY) and change rate (%) of ecological service value in the Qihe watershed from 2000 to 2015.

Land-Use	ESV/1 × 10^8^ CNY				2000–2005		2005–2010		2010–2015	
/Cover Type	2000	2005	2010	2015	Change Amount/1 × 10^8^ CNY	Change Rate/%	Change Amount/1 × 10^8^ CNY	Change Rate/%	Change Amount/1 × 10^8^ CNY	Change Rate/%
Cultivated land	5.23	4.87	4.71	4.31	−0.36	−7	−0.15	−3.12	−0.41	−8.67
Forest land	10.33	10.99	10.28	10.01	0.66	6	−0.71	−6.51	−0.26	−2.57
Grassland	2.49	2.5	2.64	2.81	−0.01	−0.4	0.14	5.6	0.17	6.43
Waters	1.46	1.47	1.65	2.28	0.0027	0.2	0.19	12.79	0.62	37.68
Unused land	0.00	0.00	0.00	0.00	0.00	0.00	0.00	0.00	0.00	0.00
Construction Land	0.00	0.00	0.00	0.00	0.00	0.00	0.00	0.00	0.00	0.00
Total	19.54	19.81	19.29	19.4	0.27	1.38	−0.52	−2.62	0.11	0.57

**Table 4:** Ecosystem service value (1 × 10^8^ CNY) and the contribution rate (%) of the Qihe watershed.

Type I	Type II	2000		2005		2010		2015		Grade
		ESV	Contribution Rate	ESV	Contribution Rate	ESV	Contribution Rate	ESV	Contribution Rate	
Adjustment Services	Gas Regulation (GR)	1.94	9.92	2.01	10.15	1.9	9.85	1.83	9.43	6
	Climate Regulation (CR)	2.05	10.49	2.08	10.5	1.99	10.32	1.92	9.9	5
	Water Conservation (WC)	2.67	13.66	2.73	13.78	2.72	14.11	2.93	15.1	3
Support Services	Soil formation And conservation (SFC)	3.11	15.91	3.14	15.85	3.01	15.6	2.89	14.9	2
	Waste Disposal (WD)	3.64	18.62	3.61	18.22	3.63	18.82	3.82	19.69	1
	Biodiversity Conservation (BC)	2.52	12.89	2.58	13.02	2.49	12.91	2.47	12.73	4
Supply Services	Food Production (FP)	1.66	8.49	1.6	8.07	1.59	8.24	1.56	8.04	7
	Raw Materials (RM)	1.17	5.98	1.24	6.26	1.16	6.01	1.12	5.77	8
Cultural Services	Entertainment Culture (EC)	0.78	3.99	0.82	4.14	0.8	4.14	0.85	4.38	9
	Total	19.54	100	19.81	100	19.29	100	19.4	100	-

**Table 5:** Changes in the total value (CNY Billion), amount of change (CNY Billion) and sensitivity index of ecosystem services in Qihe watershed after adjustment.

	ESV/(CNY Billion)				Amount of Change/(CNY Billion)				Sensitivity Index (CS)			
Value Factor	2000	2005	2010	2015	2000–2005	2005–2010	2010–2015	2000–2015	2000	2005	2010	2015
Cultivated land VC + 50%	22.13	22.25	21.64	21.56	0.12	−0.61	−0.08	−0.57	0.27	0.25	0.24	0.22
Cultivated land VC-50%	16.90	17.39	16.93	17.26	0.49	−0.46	0.32	0.36				
Forestland VC + 50%	24.69	25.32	24.43	24.42	0.63	−0.89	−0.01	−0.27	0.53	0.55	0.53	0.52
Forestland VC-50%	14.35	14.32	14.15	14.40	−0.03	−0.17	0.25	0.05				
Grassland VC + 50%	20.77	21.06	20.61	20.81	0.29	−0.45	0.21	0.04	0.13	0.13	0.14	0.14
Grassland VC + 50%	18.27	18.57	17.97	18.00	0.30	−0.60	0.04	−0.27				
Water VC + 50%	20.25	20.55	20.11	20.55	0.30	−0.44	0.43	0.30	0.07	0.07	0.09	0.12
Water VC-50%	18.79	19.09	18.46	18.27	0.30	−0.63	−0.19	−0.52				
Unused land VC + 50%	19.52	19.82	19.29	19.41	0.30	−0.53	0.12	−0.11	0.0005	0.0005	0.001	0.001
Unused land VC-50%	19.52	19.82	19.29	19.41	0.30	−0.53	0.12	−0.11				

## Data Availability

Not applicable.
